# Expression of AFP and STAT3 Is Involved in Arsenic Trioxide-Induced Apoptosis and Inhibition of Proliferation in AFP-Producing Gastric Cancer Cells

**DOI:** 10.1371/journal.pone.0054774

**Published:** 2013-01-30

**Authors:** Yanfei Jia, Dezhi Liu, Dongjie Xiao, Xiaoli Ma, Shuyi Han, Yan Zheng, Shanhui Sun, Maoxiu Zhang, Hongmei Gao, Xia Cui, Yunshan Wang

**Affiliations:** 1 Central Laboratory, Jinan Central Hospital Affiliated to Shandong University, Jinan, China; 2 Shandong Province Key Lab of Tumor Target Molecule, Jinan Central Hospital Affiliated to Shandong University, Jinan, China; 3 College of Life Science, Shandong Normal University, Jinan, China; University of Navarra School of Medicine and Center for Applied Medical Research (CIMA), Spain

## Abstract

Alpha-fetoprotein (AFP)-producing gastric cancer (AFPGC), represented by the production of AFP, has a more aggressive behavior than common gastric cancer. The underlying mechanisms are not well understood. Arsenic trioxide (As_2_O_3_) is used clinically to treat acute promyelocytic leukemia(APL) and has activity *in vitro* against several solid tumor cell lines, with induction of apoptosis and inhibition of proliferation the prime effects. Signal transducer and activator of transcription 3 (STAT3) has an important role in tumorigenesis of various primary cancers and cancer cell by upregulating cell-survival and downregulating tumor suppressor proteins. Here, we found decreased expression of AFP and STAT3 after induction of apoptosis by As_2_O_3_ in the AFPGC FU97 cells. Also, the level of the STAT3 target oncogene Bcl-2 was decreased with As_2_O_3_, and that of the tumor suppressor Bax was increased. Furthermore, STAT3 expression and depth of invasion and lymph node metastasis were associated. Survival of patients with gastric cancer was lower with AFP and STAT3 double overexpression than with overexpression of either alone. Downregulation of AFP and STAT3 expression plays an important role in As_2_O_3_-induced apoptosis of AFPGC cells, which suggests a new mechanism of As_2_O_3_-induced cell apoptosis. As_2_O_3_ may be a possible agent for AFPGC treatment.

## Introduction

Alpha-fetoprotein (AFP) is a major plasma protein produced by the yolk sac and liver during fetal life. In clinical practice, AFP is often used as a tumor marker of hepatocellular carcinoma and yolk sac tumors. Some studies have demonstrated that the other tumors in human could also produce AFP and gastric cancer was one of the most common [1]–[8]. AFP-producing gastric cancer (AFPGC) has a more aggressive behavior than common gastric cancer because the disease progresses rapidly and metastasizes frequently in the regional lymph nodes and liver [7]–[10]. Furthermore, AFPGC is associated with shorter interval-free liver metastasis after radical surgery, and the survival rate is significantly poorer with AFPGC than without AFP production [1], [9], [10]. Thus, AFP may be a passive tumor marker and an active tumor growth stimulator. Downregulating AFP expression may be an effective approach to AFP-producing cancer.

Arsenic trioxide (As_2_O_3_) has been successfully used to treat leukemia [11]–[13] and is active in several solid tumors, including gastric cancer [14]. The mechanism of action of As_2_O_3_ includes affecting the activities of Akt, JNK kinases, NF-κB, glutathione, calcium signaling, reactive oxygen species(ROS), and caspases, as well as pro- and anti-apoptotic proteins [15]–[18]. No standard chemotherapy is available for AFPGC, although some regimens have demonstrated efficacy in a small number of cases [19]–[21]. Also, the effects and mechanism of As_2_O_3_ against AFPGC remain unclear.

Signal transducer and activator of transcription 3 (STAT3) is involved in both signal transduction and transcription activation and has important roles in various biological processes such as metabolism and tumorigenesis [22], [23]. STAT3 is constitutively activated in a wide variety of cancer types [24], [25]. Targeting STAT3 may be an effective approach to addressing tumor progression. This STAT3 effect is mediated through regulation of various STAT3 target genes, including apoptosis inhibitors and cell cycle regulators, such as Bcl-2, Mcl-1, survivin, p53, c-Myc, cyclin D1 [26]–[31], and vascular endothelial growth factor (VEGF) [32]. However, the role of STAT3 in AFPGC is poorly understood.

Here, we examined the effect of As_2_O_3_ on proliferation and AFP and STAT3 expression in AFPGC FU97 cells. We evaluated downstream events of STAT3 signaling, Bcl-2 and Bax expression, to explore the possible mechanisms underlying this phenomenon. Furthermore, we evaluated the expression of AFP and STAT3 by immunohistochemistry in human gastric cancer samples. Downregulation AFP and STAT3 expression contributed to As_2_O_3_-induced apoptosis and inhibition of proliferation in AFPGC.

## Materials and Methods

### Ethics Statement

The study protocol was approved by the Medical Ethics andHuman Clinical Trial Committee of the Jinan Central Hospital. Written informed consent was obtained from all patients.

### Cell Culture and Drug Treatment

The human AFPGC cell line FU97 was obtained from the Japanese Collection of Research Bioresources (Japan) and was maintained in DMEM (Invitrogen) supplemented with 10% fetal bovine serum (FBS; Invitrogen), with 1% antibiotics at 37°C in 5% CO_2_ humidified air.

As_2_O_3_ (Sigma) was dissolved in phosphate buffered saline (PBS) at 1 mol/L as a stock solution and stored at 4°C. For *in vitro* use, the stock solution was diluted to the appropriate concentration in growth medium without FBS. Exponentially growing cells were treated with As_2_O_3_ at final concentrations of 1, 5, or 10 µmol/L. Control cultures were treated with distilled PBS at a final concentration of 0.1% in culture medium. All experiments were performed in triplicate.

### MTT Cytotoxicity Assay

The effect of As_2_O_3_ on inhibiting *in vitro* growth of FU97 cells was determined by measuring MTT (3-[4,5-dimethylthiazol-2-yl]-2,5-diphenyltetrazolium bromide) dye absorbance of living cells. FU97 cells were seeded in 96-well plates at 1.6× 10^3^ cells per well in 100 µL DMEM containing 10% FBS overnight. After exposure to various concentrations of As_2_O_3_ for 24, 48 and 72 h, 20 µL (5 g/L) MTT (Sigma, St. Louis, MO) solution was added to each well and plates were incubated for an additional 4 h at 37°C. Formazine was dissolved in 150 µL/well dimethyl sulfoxide (DMSO) and the absorbance was detected at 490 nm. Inhibitory rate (%) = (1−A value in experimental group/A value in control group) × 100%. The 0 µmol/L group was used as blank control.

### DNA Fragmentation Analysis by Electrophoresis

A total of 10^6^ cells was gently scraped from dishes, washed twice in cold PBS, and centrifuged at 15000 rpm for 10 min, then lysed in 200 µL lysis buffer (1 mL of 1 M Tris–HCl buffer, pH 7.4, 0.2 mL of 0.5 M ethylenediaminetetraacetic acid [EDTA], 0.5 mL of 10% Triton X-100). Lysed cells were held at 4°C for 10 min, and supernatants were incubated with 2 µL RNase A (10 mg/mL in Tris–EDTA buffer) at 50°C for 30 min, then with 2 µL proteinase K (10 mg/mL in distilled water) for 45 min at 50°C. The solution was mixed with 5 M NaCl (20 µL) and 2-propanol (120 µL), incubated at 20°C for 24 h, then centrifuged at 15000 rpm for 20 min. The precipitated DNA was dissolved in Tris–EDTA (5 µL) buffer and underwent electrophoresis with 2% agarose gel and Tris–acetate–EDTA buffer at 50 V. The DNA fragmentation pattern was visualized with use of a UV transilluminator.

### Hoechst 33258 Staining Analysis of Cell Apoptosis

Cells grown on the glass cover-slips were fixed with 4% paraformaldehyde/PBS for 30 min, washed for 15 min in 0.1% Triton X-100/PBS and incubated in dark with Hoechst 33258 (10 µg/ml) for 15 min. After the cover-slips were washed in PBS, positive nuclei were counted. Normal nuclei and apoptotic nuclei (condensed or fragmented chromatin) were easily distinguished.

### Quantitative Real-time PCR

Cells were cultured with As_2_O_3_ (5 µmol/L) for 72 h. Total RNA was extracted with use of Trizol reagent (Invitrogen, Carlsbad, CA, USA) and quantified by spectrophotometry. First-strand cDNA was prepared with use of random primers following the kit instructions (Takara, Japan). Real-time quantitative PCR of AFP, STAT3 and its downstream genes involved the 7300 Real-time PCR System (ABI, USA) with Takara SYBR Premix Ex Taq reagents (Takara, Japan). Primers were designed and validated by Invitrogen. The primer information is in Table 1. PCR reactions were carried out in triplicate in a 20-µL volume for 2 min at 94°C for initial denaturing, followed by 30 cycles at 94°C for 30 s and at 60°C for 45 s. A housekeeping control gene GAPDH was used as an internal control. Each primer set was first tested to determine optimal concentrations, and products were run on a 1% agarose gel to confirm the appropriate size. Subsequently, ABI dissociation curve software was used to control for multiple species in each PCR amplification. cDNA from FU97 cells without As_2_O_3_ treatment was used to construct a standard curve for each gene.

**Table 1:** Primer sequences for real-time PCR.

Gene		Primer sequence
GAPDH	Forward	GCACCGTCAAGGCTGAGAAC
	Reverse	TGGTGGT GAAGACGCCAGT
AFP	Forward	AAATGCGTTTCTCGTTGC
	Reverse	TTTCATCCACCACCAAGC
STAT3	Forward	ATCACGCCTTCTACAGACTGC
	Reverse	CATCCTGGAGATTCTCTACCACT
Bcl-2	Forward	ATGTGTGTGGAGAGCGTCAACC
	Reverse	GAGCAGAGTCTTCAGAGACAGCC
BAX	Forward	ATTGCCGCCGTGGACACAGA
	Reverse	ATGGTGAGTGAGGCGGTGAG

### Western Blot Analysis

Cell pellets were homogenized in extraction buffer (50 mmol/L Tris-HCl, pH 6.8, 0.1% SDS, 150 µmol/L NaCl, 100 mg/L phenylmethylsulfonyl fluoride, 1 mg/L aprotinin, 1% NP-40 and 0.5% sodium orthovanadate), incubated at 4°C for 30 min, and centrifuged for 20 min at 12 000 g/min. Total protein in the cell lysate was measured with use of the Bio-Rad colorimetric kit (Bio-Rad, Hercules, CA, USA). For western blot analysis, total protein was separated on 10% SDS-PAGE and transferred onto nitrocellulose membranes (0.45 µm, Millipore, Billerica, MA, USA), which were incubated for 24 h at 4°C with the antibodies for AFP (1∶500, R&D), STAT3 (1∶1000),caspase 3 (1∶500), Bcl-2 (1∶500), BAX(1∶500) and GAPDH (1∶1000; all Cell signaling technology), then horseradish peroxidase-conjugated anti-mouse/rabbit IgG antibody (Santa Cruz Biotechnology) after a final wash. Reactions were developed with use of 4-chloro-1-naphthol (Sigma) and H_2_O_2_. Signals were detected with use of an enhanced chemiluminescence kit (Amersham Pharmacia, Buckinghamshire, UK). GAPDH level was an internal standard.

### Immunoassay of AFP Concentration in Supernatant

The supernatant of FU97 cells were collected after treatment with As_2_O_3_ or negative control for 24, 48 and 72 h.AFP concentration in supernatant was determined by two-site immunoenzymometric assay in an TOSOH AIA system (Japan). The cut-off value for AFP was 10 ng/ml.

### Patients

We examined data from surgical and pathological records for 24 patients with AFPGC and 24 randomly selected patients with normal levels of serum AFP and matched to AFPGC patients by gastric cancer stage. Patients had undergone surgical resection at the Clinical Hospital of Shandong University, China, from January 1996 to December 2011. AFPGC patients showed elevated serum AFP level but no concomitant liver diseases. Histopathological presence of AFP positivity was confirmed by immunohistochemistry. We contacted each patient to confirm survival or date of death.

### Immunohistochemistry

Immunohistochemistry involved use of biotin-streptavidin-peroxidase with a Vectastain ABC kit (Vector Laboratories, CA, USA). Briefly, tissue sections (4 mm) were prepared from paraffin-embedded tissue specimens. The sections were deparaffinized with xylene followed by dehydration in graded alcohol. Sections were heated in a microwave for 2 min at 900 W to retrieve the antigen, and then incubated with 0.3% H_2_O_2_ solution in methanol for 30 min to block endogenous peroxidase. After 3 washes with phosphatebuffered saline (PBS), slides were incubated with 10% normal horse serum to block nonspecific background staining, then incubated with primary antibodies rabbit anti-AFP (1∶100 dilution) and anti-STAT3 (1∶200) in a humid chamber at 4°C overnight. After a washing with PBS, sections were incubated with biotinylated-horse anti-mouse antibodies for 30 min, washed 3 times with PBS, and incubated with streptavidin-conjugated peroxidase for 30 min. Sections were visualized by incubation with 3, 3′-diaminobenzidine solution (0.3% H_2_O_2_ and 0.05% 3, 3′-diaminobenzidine) and counterstained with hematoxylin. Omission of the primary antibody was a negative control. Every run included a positive control and a negative control. For the negative control, the primary antibody was replaced with PBS.

### Statistical Analysis

Data are expressed as mean ± SD and were analyzed by use of SPSS v11.5 (SPSS Inc., Chicago, IL, USA). The association of clinicopathologic variables and AFP and STAT3 expression was determined by chi-square test, and Yate’s correction was applied in a small number of samples. Chi-square test or two-tailed Student’s *t* test was used for assessing differences between groups. Analysis of survival involved the log–rank test, with Kaplan–Meier curves. P<0.05 was considered statistically significant.

## Results

### Growth Inhibition and Apoptosis Induction in FU97 Cells by As_2_O_3_

**Figure 1:**
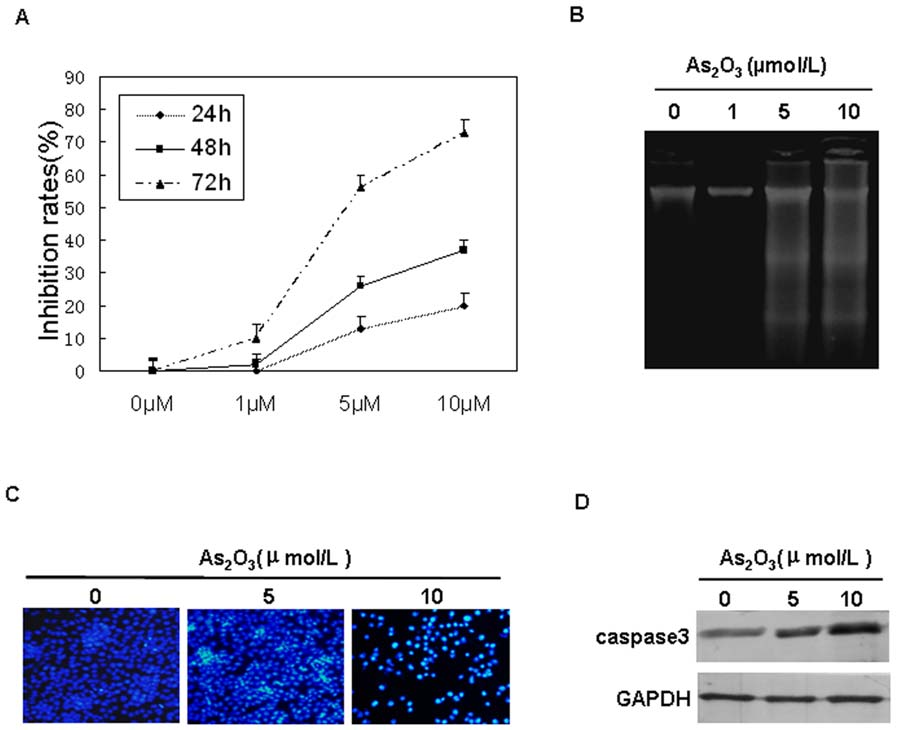
Arsenic trioxide (As2O3)-induced growth inhibition and apoptosis of gastric cancer FU97 cells. (A**)** Cellular growth inhibition measured by MTT assay. Data are mean ± SD of 3 independent experiments. (B) Agarose gel analysis of DNA fragmentation in FU97 cells treated with As2O3 for 72 h.(C) Apoptotic nuclei stained with Hoechst 33258 show intense fluorescence corresponding to chromatin condensation and fragmentation.(D) Western blot analysis of caspase3 protein in total cell extracts of FU97 cells treated with the indicated concentration of As2O3 for 72 h. GAPDH expression served as loading control.

FU97 cells were treated with different concentrations of As_2_O_3_ (1, 5 and 10 µmol/L) at 24, 48 and 72 h. As_2_O_3_ inhibited the proliferation of FU97 cells concentration and time dependently (Fig. 1A). In cells treated with 5 µmol/L and 10 µmol/L As_2_O_3_ for 72 h, the growth inhibition was 56.27±3.91% and 73.46±4.64%, respectively. DNA fragmentation is a characteristic feature of the apoptotic process. Typical DNA fragmentation ladders were detected in FU97 cells treated with 5 µmol/L and 10 µmol/L As_2_O_3_ (Fig. 1B). Here, we further study the morphological change of apoptotic cell. FU97 cells treated with 5 µmol/L and 10 µmol/L As_2_O_3_ for 72 h were stained using Hoechst 33258, and observed under fluorescent microscope.The results showed that As_2_O_3_ induced FU97 cells apoptosis in a concentration dependent manner, as indicated by fragmented and condensed nuclei (Fig. 1C). To elucidate the further influence of As_2_O_3_ on cell apoptosis, caspase3 was measured by western blot.The results from Fig. 1D clearly demonstrated that caspase 3 protein expression increased significantly in FU97 cells.Therefore, As_2_O_3_ could inhibit cell growth and induce apoptosis of AFPGC FU97 cells.

### Inhibitory Effect of As_2_O_3_ on Expression of AFP and STAT3 in FU97 Cells

**Figure 2:**
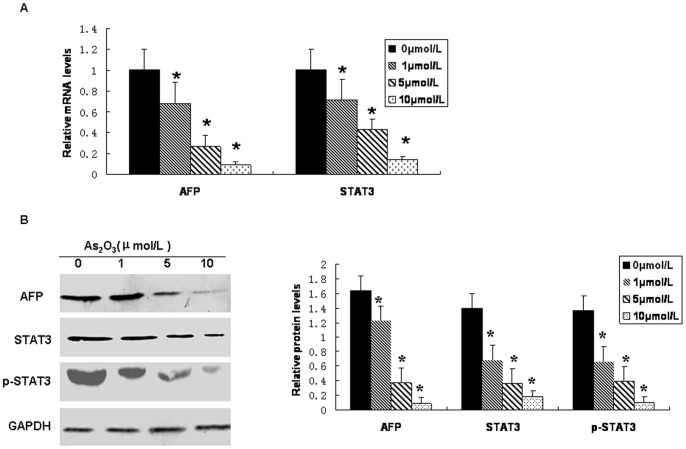
As2O3 downregulates AFP, STAT3, and phospho-STAT3 expression in FU97 cells. Cells were exposed to As2O3 at 5 µmol/L for 72 h. (A) Quantitative RT-PCR of mRNA expression. (B) Western blot analysis and quantification of protein expression. Data are representative of 3 independent experiments with similar results. *p<0.05 compared with 0 µmol/L.

To elucidate the role of AFP and STAT3 in the As_2_O_3_ induced inhibition of proliferation and apoptosis, the effect of As_2_O_3_ on AFP and STAT3 expression was examined using quantitative real-time PCR and western blot. Cells were treated with 1, 5 and 10 µmol/L As_2_O_3_ for 72 h.The mRNA and protein expression of AFP and STAT3 and phosphorylation of STAT3 were significantly downregulated with As_2_O_3_ treatment in a concentration dependent manner (Fig. 2, Fig. 7).

### AFP Concentration Associated with Growth Inhibition and Apoptosis in the FU97 Cell Culture Supernatant

**Figure 3:**
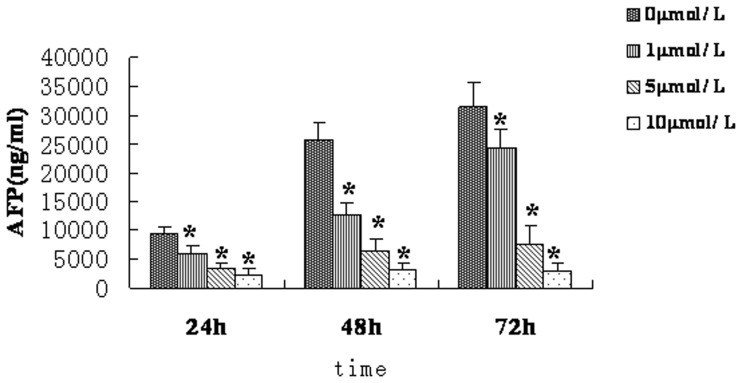
Effect of As2O3 on AFP concentrations in cell culture supernatant of FU97 cells. As_2_O_3_ decreased AFP protein level concentration dependently. Data are representative of 3 independent experiments with similar results. *p<0.05 compared with 0 µmol/L.

To further confirm the inhibitory effect of As_2_O_3_ on AFP, we measured AFP protein level in supernatant of FU97 cells. As_2_O_3_ could decrease AFP protein level concentration dependently (Fig. 3, Fig. 7). This result agreed with the cellular growth inhibition ratio (Fig. 1A), apoptosis of FU97 cells (Fig. 1B,C,D), and reduced mRNA expression of AFP and STAT3 (Fig. 2A) and protein expression of AFP, STAT3 and pSTAT3 (Fig. 2B).

### Reduced Levels of STAT3 Targeting Genes, Bcl-2 and Bax, with As_2_O_3_ Treatment in FU97 Cells

**Figure 4:**
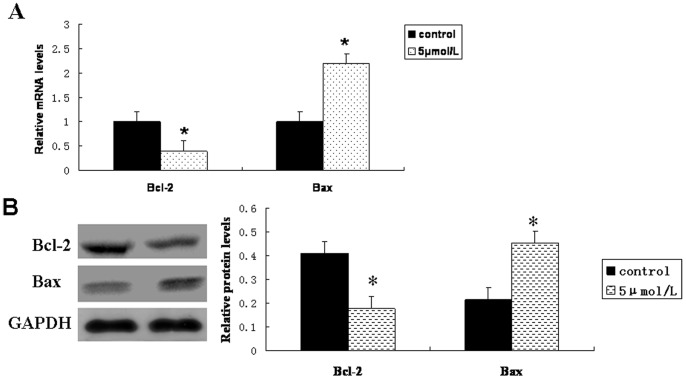
Effect of As2O3 on expression of STAT3 target genes Bcl-2 and Bax in FU97 cells. (A) Quantitative RT-PCR and (B) western blot analysis and quantification of cells treated with As_2_O_3_ at 5 µmol/L for 72 h. The mRNA and protein expression of Bcl-2 was downregulated in As_2_O_3_ treated cells, but that of Bax was upregulated. All experiments were performed in triplicates. *p<0.05 compared with control.

To explore the expression of STAT3 targeting genes, we examined the expression of anti-apoptotic Bcl-2 and pro-apoptotic Bax in FU97 cells treated with As_2_O_3_. The mRNA and protein expression of Bcl-2 was downregulated in As_2_O_3_ treated cells (Fig. 4) but that of Bax was upregulated, which suggests that the effect of As_2_O_3_ in cell apoptosis was mediated by inhibition of constitutively activated STAT3 (Fig. 7).

### Clinical Characteristics of the Selected Population

There were 34 male (70.8%) and 14 female (29.2%) patients, with a median age of 66 years(range, 45–83 years). The clinicopathological characteristics of the patients were summarized in Table 2. There were 48 patients had complete follow-up data, and the follow-up period was from 3 months to 60 months,with a mean period of 33.7 months. The overall survival time was defined as the months from the date of surgery to the date of death or loss follow-up.

**Table 2:** Association of clinicopathologic features with signal transducer and activator of transcription 3 (STAT3) expression in the primary tumor of alpha-fetoprotein (AFP)-positive and -negative gastric cancers.

	AFP(+) (n = 24)			AFP(–) (n = 24)			
	STAT3(+)(n = 11)	STAT3(–)(n = 13)	p	STAT3(+)(n = 8)	STAT3(–)(n = 16)	p	
Age							
<60	5(42%)	7(58%)		3(38%)	5(62%)		
≥60	6(50%)	6(50%)	0.68	5(31%)	11(69%)	0.76	
Sex							
Female	2(40%)	3(60%)		2(22%)	7(78%)		
Male	9(47%)	10(53%)	0.77	6(40%)	9(60%)	0.37	
Depth ofinvasion							
T1/T2	3(25%)	9(75%)		3(19%)	13(71%)		
T3/T4	8(67%)	4(33%)	0.04*	5(63%)	3(37%)	0.03*	
Pathologystage							
I–II	3(27%)	8(72%)		2(18%)	9(72%)		
III–IV	8(62%)	5(38%)	0.09	6(46%)	7(54%)	0.14	
Lymph nodemetastasis							
No	0(0%)	5(100%)		1(1%)	10(99%)		
Yes	11(58%)	8(42%)	0.02*	7(54%)	6(46%)	0.02*	

Figures in parentheses are percentages.

* Considered to be statistically significant.

### Immunohistochemical Expression of STAT3

**Figure 5:**
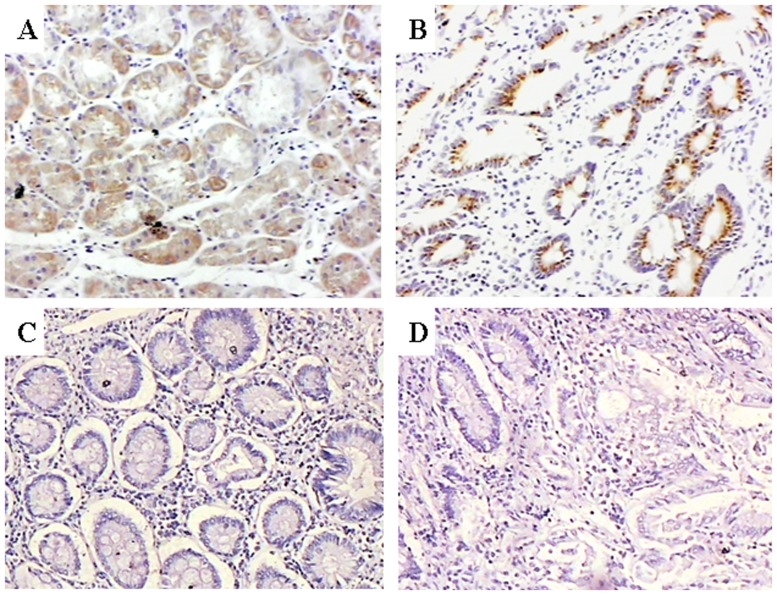
Representative immunohistochemical staining in serial sections of poorly differentiated adenocarcinoma of the stomach from patients positive for AFP (magnification ×100). (A) Immunostaining for AFP. (B) Strong STAT3 immunostaining with brown granular deposits in the cytoplasm and nuclei. (C) Negative control immunohistochemical staining for AFP. (D) Negative control immunohistochemical staining for STAT3.

Because we lack information on the expression of STAT3 in AFPGC, we determined its expression by immunohistochemical staining of AFPGC patient tissue. In the 24 AFPGC primary tumors, 11 were positive (46%) and 13 were negative (54%) for STAT3 expression. In the 24 AFP-negative gastric cancer samples, 8 (33%) primary tumors were positive and 16 (67%) were negative for STAT3 expression. Moreover, despite the relatively low numbers of patients with complete data, STAT3 overexpression was significantly associated with the depth of invasion and lymph node metastasis (p<0.05) in the AFP-positive and -negative groups (Fig. 5, Table 2, Fig. 7).

### Expression of AFP and STAT3 Associated with Poor Prognosis of Gastric Cancer

**Figure 6:**
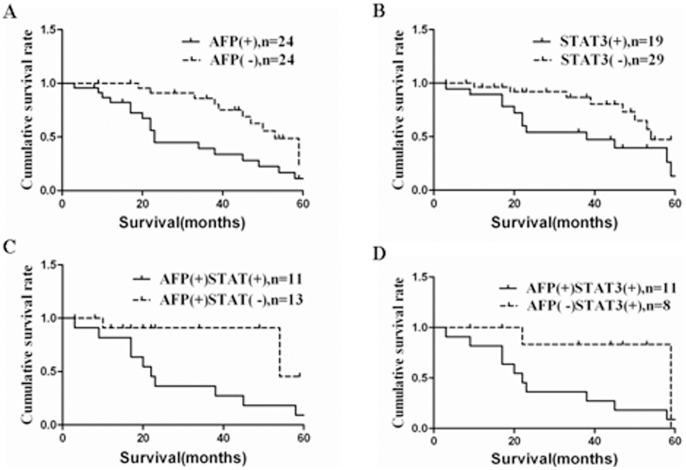
Kaplan–Meier survival curves and log-rank test for patients with AFP-positive gastric cancer (AFPGC) stratified by AFP and STAT3 expression. (**A**) AFP positivity alone. (**B**) STAT3 positivity alone. (**C**) AFP and STAT3 double positivity compared with AFP positivity. (**D**) AFP and STAT3 double positivity compared with STAT3 positivity(all P<0.05).

**Figure 7:**
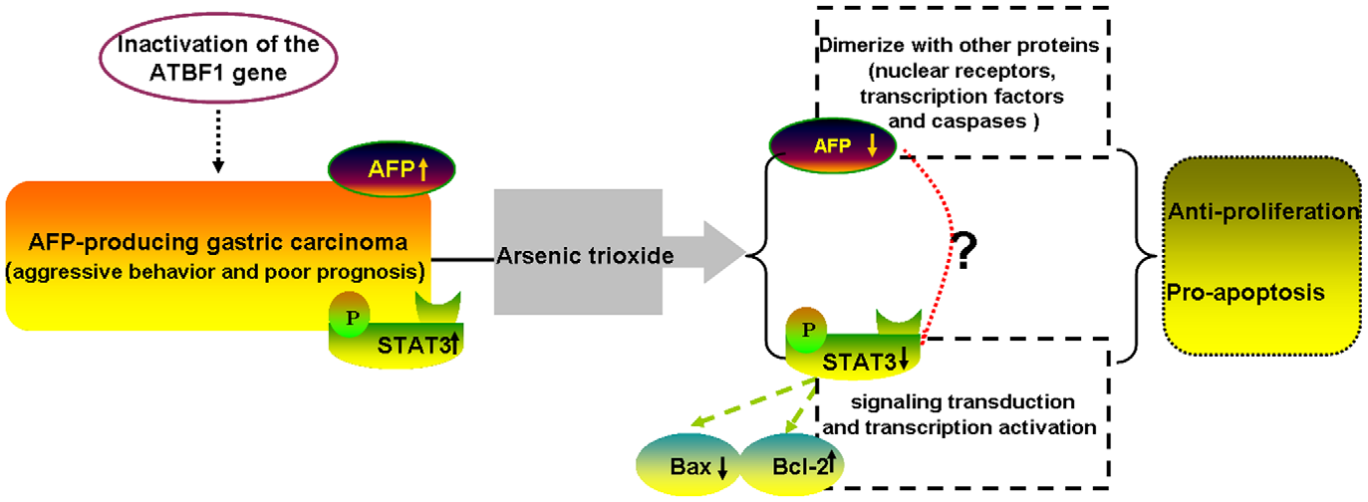
Schematic illustration of As2O3-induced growth inhibition and apoptosis of FU97 cells. Inactivation of the ATBF1 gene in AFPGC, through mutation or reduced expression, may allow AFPGC cells to produce AFP protein and overexpress STAT3, which contributes to aggressive behavior and poor prognosis of AFPGC. As2O3 can inhibit AFPGC cell growth and induce cell apoptosis. The underlying mechanisms may involve downregulation of AFP and STAT3 expression and STAT3 downregulating the expression of anti-apoptotic Bcl-2 and upregulating that of the tumor suppressor Bax. Furthermore, AFP can dimerize with other proteins such as nuclear receptors, transcription factors and caspases, all of which can promote growth of tumor cells. AFP may dimerize with the transcription factor STAT3 to promote AFPGC growth. Therefore, AFP may interact with STAT3 in the signal pathway for chemotherapeutic efficiency of agents on AFPGC.

The median survival time of AFP-positive patients was 23 months (95% confidence interval, 16–30 months). which was significantly shorter than that in the AFP-negative patients, 53 months (95% confidence interval, 47–59 months) (P<0.05).The median survival time in the STAT3-positive group was 38 months (95% confidence interval, 29–47 months), which was significantly shorter than that in the STAT3-negative group, 54 months (95% confidence interval, 47–61 months) (P<0.05, Fig. 6A and B, Fig. 7).Furthermore, survival was lower for AFP and STAT3 double-positive patients than with expression of AFP or STAT3 alone (P<0.05, Fig. 6C and D, Fig. 7). In patients with AFP and STAT3 double-positive expression the median survival time was 22 months (95% confidence interval 11–33 months). In comparison, in patients with single expression of AFP or STAT3, the median survival time was 54 months (95% confidence interval 44–64 months) and 59 months (95% confidence interval 47–71 months).

## Discussion

AFPGC has been difficult to treat mainly because of the propensity for metastasis and resistance to conventional therapies. Alternative therapies specifically addressing these issues are urgently needed. As_2_O_3_ has been used in clinical trials of APL for years, and 10 mg/day As_2_O_3_ was found effective in inducing complete remission in patients with newly diagnosed and relapsed APL [33]. As_2_O_3_ inhibited cell proliferation and induced apoptosis of the human hepatocellular carcinoma cell line HepG2 [34],which was further supported by a clinical trial showing As_2_O_3_ to be effective and safe for patients with advanced primary carcinoma of the liver [35],the toxicity was mild and serum AFP level was decreased. Therefore, we investigated whether As_2_O_3_ also induced cell growth inhibition and apoptosis of hepatoid adenocarcinoma of stomach AFPGC FU97 cells and found the antitumor properties of As_2_O_3_ in AFPGC cells.

We provide evidence that As_2_O_3_ has a strong anti-proliferative effect on FU97 cells in a concentration and time dependent manner. The plasma level of arsenic in the clinical management of acute promyelocytic leukemia can reach 5.5–7.3 mM [36]. In our study, all the results showed that 5 µmol/L As_2_O_3_ efficiently inhibited the proliferation and induced cell apoptosis at 72 h. This dosage is clinically relevant, which suggests that AFPGC cells are sensitive to As_2_O_3_.

AFPGC, as represented by the production of AFP, exhibits higher proliferative activity, weaker apoptosis, and richer neovascularization than AFP-negative gastric cancers. On the other hand, high levels of AFP in fully developed hepatocarcinoma or in serum of the host are associated with more aggressive behavior, and increased anaplasia [37], [38]. Studies of AFP knockdown by siRNA found inhibited cell proliferation in hepatomas [39]. Therefore, AFP may function in a fundamental step in the progression of AFP-positive cancer. Downregulation of AFP expression may represent a relevant therapeutic strategy. We found that As_2_O_3_ could downregulate AFP mRNA and protein expression. Also, downregulation of AFP by As_2_O_3_ could inhibit cell proliferation and induce cell apoptosis in AFPGC FU97 cells. Moreover, AFP secretion in As_2_O_3_-treated cells was dose- and time-dependently decreased in the supernatant. Downregulation of AFP expression might contribute to As_2_O_3_-induced inhibition of cell growth and apoptosis. Thus, these data indicated that AFP expression is downregulated in response to As_2_O_3_ treatment. AFP may play an important role in the proliferation and apoptosis of AFPGC.

In addition to being a point of convergence for numerous oncogenic signaling pathways, STAT3 also participates in cell growth and survival. In leukemia cells, As_2_O_3_ activates numerous intracellular signal transduction pathways, thus resulting in induction of apoptosis [40]. As_2_O_3_ inhibition of STAT3, before inhibition of cellular proliferation, has been described in multiple myeloma cells [41]. Also, As_2_O_3_ inhibits protein tyrosine kinase, thereby indirectly decreasing activation of STAT proteins [42].Therefore, downregulation of STAT3 has been considered one of the mechanisms of action of As_2_O_3_ in acute promyelocytic leukemia(APL).We found STAT3 activated in AFPGC cells, and As_2_O_3_ could downregulate STAT3 mRNA expression and STAT3 and pSTAT3 protein expression. Especially, downregulated expression of STAT3 and pSTAT3 was consistent with downregulated expression of AFP by As_2_O_3._ STAT3 might be inhibited by some factor during its activation in response to As_2_O_3._ in AFPGC. Regardless of the possible mechanisms involved in the regulation of STAT3, the high AFP expression in AFPGC might have important implications. Previous studies have also shown that AFP could dimerize with other proteins, such as nuclear receptors (i.e., retinoic receptor), transcription factors and caspases, all of which can promote growth of tumor cells [43], [44]. This, in turn, has led to speculation that AFP could dimerize with transcription factors STAT3 to promote AFPGC growth. Further investigations of the regulation of STAT3 expression related to AFP are needed.

STAT3 may induce cell apoptosis by transcriptionally downregulating Bcl-2 expression [45]. Bcl-2 is an upstream effector molecule in the apoptotic pathway and a potent suppressor of apoptosis. It can oligomerize Bax, which subsequently depolarizes the mitochondrial membrane potential to release cytochrome c and induce apoptosis [46]. The ratio of Bcl-2 to Bax is important in determining whether the cells will undergo apoptosis or survival. We found reduced expression of Bcl-2 along with inhibited STAT3 expression with As_2_O_3_ treatment in AFPGC cells. In contrast, the expression of Bax was increased. Along with findings in other cell systems, where inhibited STAT3 level was found to decrease Bcl-2 expression and induce apoptosis [45], the As_2_O_3_-induced effects we found may be mediated by inhibition of constitutively activated STAT3.

To further demonstrate whether STAT3 plays a key role in AFPGC, we examined AFPGC patients in terms of STAT3 expression. STAT3 positivity in AFP-positive tumors was 46% and in AFP-negative tumors 33%. Patients with AFPGC had a higher rate of STAT3 expression, although not significant perhaps because of a small number of patients. However, STAT3-positive expression was associated with cancer tissue, depth of invasion and lymph node metastasis in AFPGC. Clinically, patients with AFPGC have poor prognosis [1], [9], [10]. We confirmed that the prognosis was worse for patients with than without AFP who were matched by cancer stage. Furthermore, the survival of patients with both AFP and STAT3 positivity was significantly worse than those with AFP or STAT3 positivity alone. Thus, in this specific type of gastric cancer, STAT3 appears to have an important role in cell survival and proliferation. STAT3 expression in AFPGC may explain the clinically aggressive behavior of AFPGC, and STAT3 expression may be a useful progression indicator, if validated by extensive prospective studies in larger cohorts. Downregulation of AFP and STAT3 expression may represent a relevant therapeutic strategy in AFPGC. Regarding the relationship between AFP production and STAT3 expression, there is no available report describing the underlying mechanisms to date.It has been reported that the AFP expression in gastric cancer is due to the lack of the transcription factor ATBF1 [47]. In addition,ATBF1,as a tumor suppressor gene, enhancesthe suppression of STAT3 signaling by interaction with PIAS3 [48].Therefore, it is reasonable to consider that inactivation of the ATBF1 gene in AFPGC,through mutation or reduced expression, may be allow AFPGC cells to produce AFP protein and overexpres STAT3. To clarify which factors are involved in AFP production and STAT3 expression,further study is needed.

In conclusion, As_2_O_3_ can inhibit AFPGC cell line FU97 growth and induce apoptosis. The possible mechanisms were related to downregulation of AFP and STAT3 and STAT3 targeting anti-apoptotic gene Bcl-2 and upregulating the tumor suppressor gene Bax (Fig. 7). The expression of STAT3 in AFPGC plays an important role in tumour invasion and prognosis. As_2_O_3_ may be a possible new adjuvant drug in treatment of AFPGC. The present study provides some theoretical basis for its clinical use worthy of further study.
